# Proposing a neural framework for the evolution of elaborate courtship displays

**DOI:** 10.7554/eLife.74860

**Published:** 2022-05-31

**Authors:** Ryan W Schwark, Matthew J Fuxjager, Marc F Schmidt

**Affiliations:** 1 Department of Biology, University of Pennsylvania Philadelphia United States; 2 Neuroscience Graduate Group, University of Pennsylvania Philadelphia United States; 3 Department of Ecology, Evolution, and Organismal Biology, Brown University Providence United States; University of Maryland School of Medicine United States; Harvard University United States

**Keywords:** display evolution, periaqueductal grey, sexual selection, neurophysiology

## Abstract

In many vertebrates, courtship occurs through the performance of elaborate behavioral displays that are as spectacular as they are complex. The question of how sexual selection acts upon these animals’ neuromuscular systems to transform a repertoire of pre-existing movements into such remarkable (if not unusual) display routines has received relatively little research attention. This is a surprising gap in knowledge, given that unraveling this extraordinary process is central to understanding the evolution of behavioral diversity and its neural control. In many vertebrates, courtship displays often push the limits of neuromuscular performance, and often in a ritualized manner. These displays can range from songs that require rapid switching between two independently controlled ‘voice boxes’ to precisely choreographed acrobatics. Here, we propose a framework for thinking about how the brain might not only control these displays, but also shape their evolution. Our framework focuses specifically on a major midbrain area, which we view as a likely important node in the orchestration of the complex neural control of behavior used in the courtship process. This area is the periaqueductal grey (PAG), as studies suggest that it is both necessary and sufficient for the production of many instinctive survival behaviors, including courtship vocalizations. Thus, we speculate about why the PAG, as well as its key inputs, might serve as targets of sexual selection for display behavior. In doing so, we attempt to combine core ideas about the neural control of behavior with principles of display evolution. Our intent is to spur research in this area and bring together neurobiologists and behavioral ecologists to more fully understand the role that the brain might play in behavioral innovation and diversification.

## Introduction

Courtship displays are ubiquitous across vertebrate taxa, varying in complexity across species from seemingly simple to highly elaborate (Hebets and Papaj, 2004; Mitoyen et al., 2019). In all cases, courtship involves movement that requires precise neuromuscular control (Fusani et al., 2014), whether it be movement of vocal musculature or acrobatic postural displays. Even iconic displays that seem rather static in nature can incorporate elaborate movement; for example the tail display of the male peafowl, which not only showcases extravagant plumage, but also involves ‘tail-rattling’ that makes the gaudy feathers shimmer in the light (Dakin et al., 2016; Petrie et al., 1991). Importantly, a growing number of studies indicate that these displays engage an animal’s neuromuscular system close to its limits, such that evaluators (i.e. receivers) become primed to use this behavior as a readout of a potential mate’s condition or genetic quality (Byers et al., 2010; Fusani et al., 2014). In many songbirds, for instance, songs that are preferred by females approach the limits of vocal performance constraints by optimizing both syllable repetition rate and acoustic bandwidth (Goller, 2022; Podos, 2001). Because courters (i.e. signalers) producing the best displays are favored, courtship routines are often driven by strong sexual selection (Andersson and Iwasa, 1996; Majerus et al., 1986; Mendelson et al., 2014; Ryan, 1998). In some cases, as long as these displays do not directly hamper the courter’s survival, such selection can drive their evolutionary elaboration into seemingly bizarre and unreal forms (Ryan, 1998; Rosenthal, 2017). Making this process even more intriguing is the fact that evaluators of a display sometimes participate in its performance by coordinating body movement with that of the courter (Barske et al., 2015; Clifton et al., 2015; Perkes et al., 2019; Slater and Mann, 2004; Soma and Iwama, 2017). Accordingly, we would expect that sexual selection acts in both the courter *and* the evaluator to drive the evolution of such behavior and the mechanisms that are necessary to support it.

**Figure 1.**
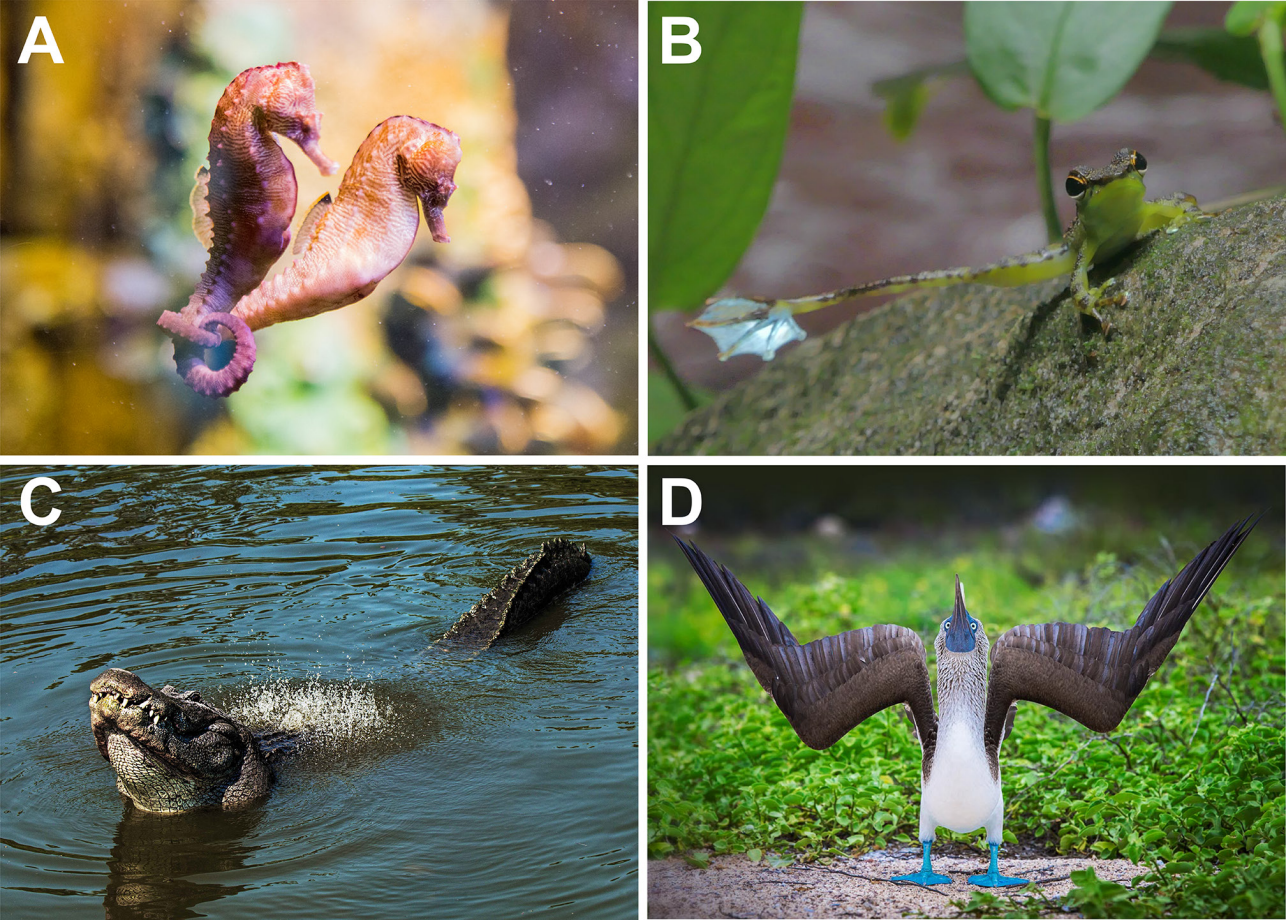
Variety of representative courtship displays among the vertebrates. (**A**) Seahorses engage in a complex, multi-phase mating ritual, which can involve rapid body vibration, changes in color, and intertwining body movements. Photo credit: Soumit Nandi, with permission. (**B**) Foot-flagging frogs extend their hindlimbs in a slow sweeping motion, with their digits spread apart to display foot webbing. Photo credit: Nabeshima Seiichiro, with permission. (**C**) Alligators perform the ‘water dance’ display, whereby deep laryngeal bellows create a striking vibratory pattern of water droplets. Photo credit: Stephen Tabone, with permission. (**D**) The blue-footed booby engages in a unique postural display which involves a slow spreading of the wings and an upward tilt of the head. Photo credit: Scott Davis, with permission through Tandem Stock. These images are not covered by the CC-BY 4.0 licence and further reproduction of these panels would need permission from the copyright holder(s).

Although some courtship behavior might be controlled primarily by a specific set of muscles — as in toadfishes where vocal emissions can be concentrated to a small group of sonic muscles responsible for vibrating the swim bladder (Bass and McKibben, 2003; Ladich and Fine, 2006) — in many animals, these behaviors engage motoneurons and muscle systems at all levels of the body. Examples can be found throughout much of the animal kingdom, ranging from the coordinated copulatory displays of stickleback fish (Kraak et al., 1999) to the strange chasing displays of bison (Lott, 2002). As shown in Figure 1, these displays are typically visually complex and consist of unique postural and gestural components. In all of these taxa, courtship displays often require exquisite coordination of a broad range of motor systems, from brainstem motoneurons that control phonatory output (Adam et al., 2021; Suthers and Margoliash, 2002) to those in both rostral and caudal spinal cord for the production of complex limb and body movements (Korn and Faber, 1996). For many species, therefore, neural control of courtship behavior will require a system that allows precise temporal coordination of many different muscle groups.

## Courtship displays as a strategy for neuromuscular assessment

Despite the seemingly endless variety of courtship behavior, all displays have been shaped by evolution for the purpose of impressing and/or attracting a mating partner. In many species, courtship displays have therefore evolved implicit mechanisms for communicating fitness information. Such information can be gathered by the physical size or strength of an individual (Rueger et al., 2016), or simply by the quality of a colorful ornament (Andersson, 1971; Veit et al., 2003). Unhealthy or stressed animals, for example, will show lower quality features (Eeva et al., 2009; Witter et al., 1997). In some cases, such as in the bowerbird, courtship displays might even include challenges that assess the individual’s cognitive abilities (Patricelli et al., 2003). In every case, however, movement forms a critical part of courtship display, and the courter is often tasked with producing behavior that poses some form of challenge to motor control. The nature of this challenge is difficult to define operationally, but it broadly refers to an individual’s ability to execute a series of precise movements that are otherwise difficult to produce. Facets of an individual’s developmental integrity, health status, or even condition, may influence how a display is performed, providing evaluators with information about the signaler upon which they can base decisions regarding mate choice (Andersson and Simmons, 2006; Guilford and Dawkins, 1991; Kirkpatrick, 1982). In theory, a lower quality individual will be unable to withstand the costs linked to optimal display performance (Grafen, 1990; Johnstone, 1995), whether those costs are related to the ability to eat the right food, out-compete one’s rivals, or withstand the negative effects of hormones necessary for display activation.

For humans, skiing provides an easy way to conceptualize this phenomenon. Many skiers can perform well when traversing down a well-groomed slope, but we can quickly find the ‘low quality’ skiers if we look at these same individuals coming down an icy mogul run. Within the animal kingdom, the ability of courtship displays to show off neuromuscular prowess is probably best illustrated in the many species of manakin (family: Pipridae) native to Central and South America (Fusani et al., 2014; Fuxjager et al., 2022; Fuxjager and Schlinger, 2015b; Schlinger et al., 2013). Males in this passerine bird family are endowed with elaborate plumage ornamentation and engage in complex mating dances. In *Manacus* manakins, one of the best-studied genera of the family, males perch on vertical twigs and rapidly jump from branch to branch at dizzying speeds, often while performing 180° acrobatic flips, all whilst producing songs and snapping their wings mid-air to generate loud popping noises (Barske et al., 2014; Chapman, 1935). Not only is each jump sequence astonishing by itself, but the birds repeat this sequence multiple times in rapid succession during the full display. Although not always as dramatic as in the manakin, many animals perform vocal-motor sequences as part of their courtship displays that push motor extremes in a way that tests coordination, speed, strength, and endurance (Clark, 2012; Draganoiu et al., 2002; Hebets and Uetz, 2000; Miles and Fuxjager, 2018a; Vliet, 1989). In many songbirds, for example, sound is produced by blowing air through the syrinx, located in the thoracic cavity at the junction of the trachea and the primary bronchi (Goller, 2022). The two side-by-side compartments of this multi-muscular vocal organ can be independently controlled by the nervous system, resulting in courtship song, for instance, that may consist of acoustic elements produced separately by each half. Remarkably, song syllables to which females pay close attention consist of independent sounds produced in each syringeal half in rapid succession (Suthers et al., 2012). In some species, the switch rate suggests that muscles gating airflow in each half must open and close within a 50-ms cycle, which is astonishingly fast (Schmidt and Goller, 2016; Suthers and Goller, 1997). Not surprisingly, muscles involved in vocalization are amongst the fastest known among vertebrates (Elemans et al., 2008).

Of course, precise neuromuscular coordination and control is not restricted to courtship displays, given that most species’ survival depends on the organism’s ability to find food and escape predators. Natural selection has, therefore, selected for behavioral traits that augment these abilities. In doing so, motor systems have been sculpted to generate many of the same ingredients of performance, such as speed, strength, endurance and coordination, that constitute a courtship display. The well-characterized C-shaped escape response of many fish species is a perfect example, being the product of coordinated activity of body, tail, and fin muscles to produce an escape response within less than 50 milliseconds (Domenici, 2010). Courtship display may therefore represent a reconfiguration of motor skills that are often already present in the animal’s ‘tool kit’. In humans, the best analogy of this concept may come from gymnasts, who can produce an extraordinary floor routine that contains many aspects of normal human locomotory behavior (e.g. running, jumping, and grasping) (McNitt-Gray et al., 2001). However, this behavior is not produced by itself, but rather is incorporated into intricate flips, twirls, and other maneuvers that require exquisite balance, coordination, strength and speed. Gymnasts, therefore, do not possess super-human performance ability; instead, they are masters at reconfiguring the fundamental performance skills that all humans are born with. For display evolution, we might then think of natural selection as the force that drives the emergence of these fundamental performance traits or attributes, whereas sexual selection is likely the force that puts them together into an unorthodox routine for the purpose of courtship. At the same time, we must also recognize the many cases in which sexual selection either produces new skills or drives evolution of existing performance skill-sets to extraordinary ends (Clifton et al., 2015; Fuxjager et al., 2016; Hogan and Stoddard, 2018).

A framework for thinking about how complex new displays might emerge during the course of evolution suggests a multistep process, known as ritualization, where inadvertent cues become co-opted into a signal or display (Bradbury and Vehrencamp, 2011; Lorenz, 1966; Tinbergen, 1952). Here, the signal precursors tend to have no communicative functions; locomotion and respiration are prime examples of a ‘behavioral template’ that can be transformed into a signal. The ritualized signal then becomes decoupled, in a process known as emancipation, from the factors that triggered it in the first place, and instead becomes coupled to novel factors that trigger its production, such as those factors associated with sociosexual contexts. Most who have studied ritualization have done so through an ethological lens, but in reality, ritualization is a neurobiological phenomenon. In other words, ritualization at its heart describes how existing neural programs that underlie ancestral behavioral conditions are transformed by selection to generate a ‘new’ display. This framework provides a critical perspective in which to examine candidate brain areas that might enable the emergence of these complex behaviors.

## Evolutionary diversity of courtship displays

**Figure 2.**
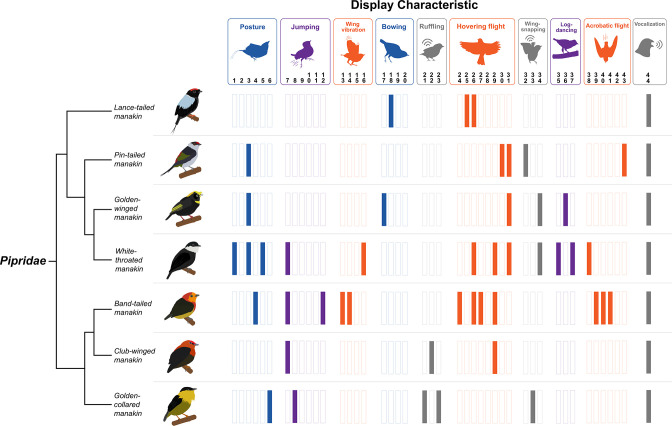
Diversity of dance display characteristics among a small group of manakin birds (family: Pipridae). Each bar represents a specific motor element that is incorporated into the given species’ (shown on the left) overall courtship routines. Each colored bar indicates that the specific element forms part of the bird’s display. The display elements are shown above and loosely categorized into different classes of behavior (e.g. posture, jumping, wing vibration, etc.). We color-coded these categories to also reflect display movements that are associated with gesture and posture (blue), forelimb locomotion (orange), hindlimb locomotion (purple), and generation of acoustic signal (grey). Note that each particular display element is denoted by a number, which corresponds to the original classification by Prum, 1990. Importantly, each bird performs a dramatically different display routine, which incorporates various forms of movement that engage different muscular systems across the body. Species’ displays also differ markedly in terms of their overall complexity, with some species performing displays with few distinct maneuvers and others displaying several distinct elements. Phylogenetic relationships from Leite et al., 2021. Bird illustrations by Ryan Schwark.

Perhaps as remarkable as their dazzling appearance, one of the most extraordinary aspects of courtship displays is their diversity across the animal tree of life. Take the manakins again as an example: while *Manacus* manakins perform the spectacular dance described above, members of their sister genus, *Heterocercus*, do something profoundly different but equally astounding. These birds perform bullet-flight displays, in which they ascend hundreds of meters above the jungle canopy and then rapidly shoot down to the forest floor, while simultaneously producing a loud conspicuous hissing sound (Prum et al., 1996). As shown in Figure 2, the diversity of manakin displays is extensive, with taxa in each genus performing a dance that is almost entirely unique unto itself (e.g. moonwalking on horizontal perches, cooperative bouts of leap-frog, backflips that occur faster than the human eye can detect, etc.; Prum, 1990; Kirwan and Green, 2012). Such diversity is by no means exclusive to manakins; rather, we see remarkable examples of radical differences in display behavior in several major lineages, including alligators, ungulates, and frogs (Caro, 1994; Garrick and Lang, 1977; Hodl and Amezquita, 2001; Ord et al., 2001). Moreover, we also see such variation among closely related taxa, including many species in select families of birds, lizards, and anurans (Anderson et al., 2021a; Miles et al., 2018c; Miles et al., 2020; Ord et al., 2001; Randall, 1997).

So, how might this diversity arise during the course of evolution? Answers to this question are complicated, and thus involve a complex set of dynamics (e.g. evolutionary history of the signaler, perceptual biases in the receiver, etc.). Still, this question has intrigued organismal biologists for decades, and it becomes even more intriguing when considered in the context of the nervous system, because the brain and its motor centers must evolve alongside these display routines to accommodate the emergence of new movement programs. In this way, we can consider how species differ in elaborate display behavior and tie this to the concomitant changes in the neural scaffold. For example, there have been suggestions that divergence in sexual display behavior occurs relatively rapidly, compared to the evolution of other traits (Martins et al., 1998; Uy and Borgia, 2000). If so, then neural and muscular systems underlying display divergence must similarly evolve on a rapid timescale. Likewise, others have suggested that the evolution of display behavior is highly labile, in that just about anything can evolve (Kusmierski et al., 1997; but see Wiens, 2000). This would suggest that neural evolution—at least that which needs to occur to support species’ differences in display behavior—must be similarly labile. Of course, we do not yet have a strong sense of whether these assumptions are the proper ones to make (they may not be).

Critical to understanding the brain’s relationship to the evolution of display (and to behavioral evolution more broadly) is the identification of neural loci that likely underlie the output of courtship display. Such knowledge will help us better predict where in the nervous system selective pressures will act, providing insight into how evolution supports behavioral divergence and innovation. In the context of motor control for display performance, we can think of these loci as the parts of nervous systems that coordinate and actuate movement. This includes a set of targets that likely extend caudally from the forebrain all the way down to lower motoneurons and the muscles they innervate, with changes to the functionality of any one of these loci helping to generate behavioral variation that acts as the raw material that fuels the evolution of display.

## Searching for a conserved neural substrate for the regulation of courtship displays

Given the complexity and diversity of courtship behavior across taxa, one could ask whether there might even exist a neural circuit or brain area whose function is dedicated to the production of courtship displays. On one hand, we know that these behaviors can be triggered by different sensory modalities (e.g. song from another bird Brenowitz, 2021; Langmore and Mulder, 2010; electric field from a neighboring fish [Hagedorn and Heiligenberg, 1985; Hopkins, 1988]) and that the displays themselves vary tremendously in the types of muscle synergies that need to be engaged. In some species, courtship might consist primarily of acoustic displays, whereas in others it might engage nearly all the muscles in the body plan. At a superficial level, we might therefore expect that each of these different behaviors is controlled by its own dedicated brain area or neural circuit. On the other hand, while the specific motor output might vary, courtship is a unified behavioral category whose ultimate purpose is linked to reproductive success (Mitoyen et al., 2019). Thus, across all taxa, we might expect the circuits controlling courtship to share several fundamental commonalities. For instance, we expect that these circuits should be under strong hormonal regulation, particularly from sex hormones (Crews and Moore, 1986). We also expect that initiation of these displays will be tightly gated and strongly context-dependent. Furthermore, the resultant behavioral output should be subjected to strong sexual selective pressures (Fuxjager and Schlinger, 2015b). Finally, these behavioral outputs should consist of precisely coordinated motor sequences that represent, in some ritualized fashion, the motor performance abilities of the individual (Byers et al., 2010). These shared properties make it reasonable to hypothesize that initiation of these behaviors is controlled by activation of a brain area that contains the action-selective code for the production of specific courtship displays. Such a center should also be gated by the specific sensory signals intended to drive courtship display and integrate information about signal valence, reproductive state, motivation, and social context.

## The midbrain PAG is necessary and sufficient for the production of courtship vocal displays

While much is still to be learned about the neural circuitry underlying the most complex courtship behaviors (e.g. those produced by the manakins), much progress has been made in our understanding of the neural control of acoustic displays (Barkan and Zornik, 2020; Ladich and Winkler, 2017; Suthers et al., 2004). In fact, close examination of the neural underpinnings of acoustic courtship provides important hints about mechanistic principles that can be applied to elaborate courtship behavior more broadly. The wealth of research into acoustic displays can be attributed to several practical factors, such as the ease with which behavior can be quantified (one needs only a microphone to measure a complex vocal display routine) and the ability to then assess how specific neuronal populations or brain areas might be causally linked to the various acoustic features in the signal. This approach has been used to decipher the neural control systems in a range of species, from the midshipman fish, which vibrate their swim bladders to produce sounds that attract females (Bass and McKibben, 2003; Ladich and Fine, 2006), to songbirds, which have an elaborate forebrain control circuit, known as the ‘song system’ (Nottebohm et al., 1976; Sakata et al., 2020; Schmidt and Martin Wild, 2014). We now also have a clear understanding of the neural circuits involved in vocal production in a range of primates (Jürgens, 2009) and are beginning to grasp the brain circuits involved in the production of ultrasonic vocalizations (USVs) used during courtship interactions in both rodents (Chen et al., 2021; Tschida et al., 2019) and bats (Salles et al., 2020; Weineck et al., 2020).

Despite the phylogenetic distance among these vertebrate taxa, all these acoustic control circuits contain a major projection to an area in the midbrain called the periaqueductal grey, or PAG (with perhaps the exception of frogs, see discussion below). This area is particularly intriguing as a possible candidate for the production of courtship displays because of its critical role in the production of innate survival behaviors and because of its role in integrating inputs from hypothalamic areas involved in sexual motivation (Silva and McNaughton, 2019). Studies dating back to the early 1900s have shown that stimulation of this area (often referred to as ‘central grey’ in earlier studies) elicits spontaneous vocalizations in birds (Brown, 1965), monkeys (Brown, 1915; Jürgens, 2002), bats (Fenzl and Schuller, 2005), and fish (Kittelberger et al., 2006). Depending on the precise site of stimulation, vocalizations can range from agonistic (e.g. growling, hissing and screaming) to non-agonistic (e.g. meowing in cats) (Subramanian et al., 2008). Furthermore, lesions to this area typically result in mutism (Bazett and Penfield, 1922). Anatomically speaking, the PAG is a highly conserved area that surrounds the cerebral aqueduct in mammals (hence its name) and extends significantly along the rostrocaudal axis (Kingsbury et al., 2011; Silva and McNaughton, 2019). It is divided into often functionally separate, but interconnected, columns that fan out from the most dorsal part of the PAG to its most ventral portion (Silva and McNaughton, 2019). Dorsal regions of the PAG act as the common motor output node for many instinctive survival behaviors, while different regions (or columns) within the dorsal PAG appear to contain action-selective codes for each of these different behaviors (Yu et al., 2021). For the remainder of the review, we will largely focus on the dorsal PAG.

**Figure 3.**
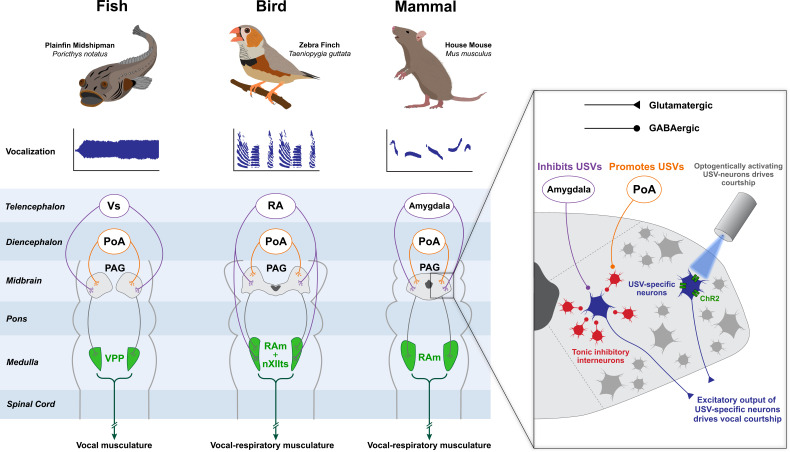
The neural circuitry governing vocal display is highly conserved among vertebrates. Anatomical and functional studies have revealed that fish, songbirds and mammals contain a conserved brainstem module which orchestrates vocal display. The role of the midbrain PAG is central, as it receives inputs from diencephalic and telencephalic structures that encode sociosexual information and in the case of songbirds even a sparse temporal code that helps shape song spectro-temporal features. Excitatory axons from the PAG then project to premotor nuclei located in the medulla, which then ultimately project to the motoneurons that innervate the vocal musculature. In the midshipman fish, vocalization is controlled by a vibratory swim bladder; in songbirds, birdsong is controlled by the muscles of the syrinx and those of respiration; in the mouse, ultrasonic vocalizations (USVs) are produced by laryngeal and respiratory muscles. The mouse PAG contains courtship-specific excitatory neurons which are necessary and sufficient to produce USVs (inset) (Tschida et al., 2019). These neurons are tonically inhibited by GABAergic interneurons, and disinhibition of the neurons by inputs from the preoptic area (POA) engages courtship. In the songbird, respiratory (RAm) and vocal (nXIIts) targets of PAG are separate anatomical structures but are combined in this schematic for simplicity. Animal illustrations by Ryan Schwark.

As shown in Figure 3, the overall position of the PAG as a common output node in these vocal control circuits (and especially in the production of acoustic signals involved in courtship behavior) seems to be remarkably conserved across phyla, while a recent study by Tschida et al., 2019 provides conclusive evidence that the PAG is necessary and sufficient for the production of vocal courtship displays in mice. Using genetic tagging of neurons that are active during the production of courtship USVs, the authors specifically showed that a subset of neurons in the dorsolateral column of the caudal PAG can be reactivated (using optogenetic approaches) to elicit USVs (Figure 3**, inset**). Further, they used inactivation (or deletion) of these same neurons to completely suppress the ability of these same mice to produce USVs during appropriate social (reproductive) contexts. Importantly, inactivation of these ‘courtship-specific neurons’ in the PAG did not cause a general suppression of vocal production, implying that this subset of PAG neurons was specifically dedicated to the production of courtship calls.

Tschida et al., 2019 also showed that these USV-specific cells have as their major target a rostrocaudally-oriented column of premotor neurons located in the ventrolateral tegmentum of the caudal medulla, known as the nucleus retroambiguus (RAm; also sometimes referred to as NRA) (Tschida et al., 2019). In mammals and birds, RAm is known to target vocal-respiratory motoneurons in the brainstem and spinal cord, and in mammals stimulation of RAm is sufficient to elicit vocalizations (Subramanian and Holstege, 2013). RAm appears to be anatomically ancient, and its role in the production of vocal signals likewise seems to be deeply conserved. Experiments in midshipman fish (family: Batrachoididae) have shown that the vocal pre-pacemaker nucleus (VPP) in the caudal medulla, which is necessary for courtship sound production, shares the same anatomical location, developmental origin and function as RAm in mammals, suggesting these two regions might be homologous (Bass et al., 2008; Bass and Chagnaud, 2012). The theme of deep conservation extends to the aforementioned PAG, which forms part of the vocal control circuit in the midshipman and is also present in lamprey, suggesting that it has been present in the vertebrate lineage for at least 560 million years (Olson et al., 2017). Although we can only speculate about the primeval function of the PAG in courtship display, in extant vertebrates it plays a strikingly homologous role in coordinating vocal courtship displays. Despite these likely homologous roles, the exact role and positioning of the PAG within different vocal control systems might vary (Figure 3). In rodents, the vocal motor circuit for USV courtship behavior seems to converge on the PAG, which acts as the final common motor output node in the circuit. PAG’s positioning within the songbird vocal control system, however, might be more nuanced. For the production of calls, PAG appears to act as the common motor output node of the circuit. By contrast, during the production of complex songs controlled by the ‘song system’, the PAG might serve as a key motor output node of the circuit in combination with brainstem vocal-respiratory centers that also receive direct input from these higher order inputs (see below for a more thorough discussion of this point).

## The PAG as a putative brain center for the generation of courtship displays

Thinking beyond just vocal displays, we hypothesize that the PAG is important to the production of many other types of courtship displays that involve body movements and gestures. There is, of course, little direct evidence that clearly supports this view, and indeed the neural basis of such display behavior remains poorly understood. As such, our ideas are largely grounded in indirect evidence that otherwise points to the PAG as a major brain region that potentially drives motor programs that make up complex courtship routines. Foremost among this evidence is the fact that, in most vertebrate systems studied to date, the PAG is described as the primary motor output node for the regulation of instinctive and relatively complex survival actions, including forms of attack and defensive behavior, hunting, parenting, mating, and vocalization (Evans et al., 2018; Falkner et al., 2020; Subramanian et al., 2008; Tovote et al., 2016; Tschida et al., 2019; Wei et al., 2021; Yu et al., 2021). A distinctive property of the PAG is that it is subdivided into molecularly and anatomically conserved subdivisions, or columns, that are tightly associated with the production of these distinct types of behavior (Bandler and Keay, 1996; Bandler and Shipley, 1994). As shown elegantly by the Tschida et al., 2019 study in the context of vocal production, each of these columns is likely to contain a localized subpopulation of neurons that is both necessary and sufficient for driving each of these behaviors. Given strong hypothetical predictions that survival and instinctive behavior, or in some cases reflexes, becomes ritualized to form exaggerated display routines (see below), it seems probable that individual motor modules of the PAG columns are critical to this process. Known interconnectivity between columns (Jansen et al., 1998) even suggests the hypothesis that under the proper context, individual modules could be ‘strung’ together to produce more complex sequences of behaviors. Again, we recognize that there is little direct empirical support for this idea, but recent work looking at the neural control of predatory hunting in mice seems to suggest it is possible, or perhaps even likely. Hunting in mice involves sequential activation of three discrete stereotyped behaviors – chase, attack, and eating. Here, the authors of this study show that each motor action is controlled by a distinct subpopulation within the dorsolateral PAG and that behavioral-specific neuronal clusters are activated in sequential order during the stereotyped hunting sequence (Yu et al., 2021). In this way, neural ensembles within the PAG itself appear to link different discrete motor acts into a more complex behavioral trait.

A hallmark feature of the PAG is its ability to integrate the many inputs it receives from key brain areas that (at least in mammals) include the amygdala, hypothalamus and areas of the pre-frontal cortex (O’Connell and Hofmann, 2011; O’Connell and Hofmann, 2012; Silva and McNaughton, 2019). These inputs likely play a critical role in regulating the drive (hypothalamic areas) and the suppression (amygdala) of instinctive behavior, ensuring that they are produced in the appropriate context. As such, it has been proposed that the PAG uses this information to ‘gate’ its activation (Jürgens, 2002; Tschida et al., 2019). In addition to acting as a gate switch, there is also growing evidence that some of these inputs might also play a role in shaping the features of these behaviors (e.g. intensity, duration) (Chen et al., 2021; Wei et al., 2021). These inputs are therefore likely to contribute in some way to the production and regulation of courtship display, and it is even conceivable (perhaps likely) that some of them serve as targets of selection.

For the remaining sections of this review, we expand upon the possibility that the PAG is a conserved brain region that likely facilitates motor control of elaborate courtship displays across vertebrates. Through this exploration, we argue that the PAG is a major site on which sexual selection can act to drive the diversification of such behavior, leading to much of the spectacular diversity in the courtship phenotype that we describe above. Of course, we appreciate that our ideas are speculative and we also acknowledge that the PAG is part of several larger brain circuits that underlie behavioral control, including courtship behavior. This means that the PAG itself is not the only brain area to potentially evolve as a means to foster display diversification, and thus we discuss other brain regions that provide critical input to the PAG, such as the preoptic area (POA) and medial amygdala, as other possible targets of selection when it acts on display behavior. Still, the PAG does seem a likely candidate to help direct the motor control of courtship display behavior (see paragraphs above), and we explore it in this spirit. Our intention is to begin developing a framework for thinking about neural control of elaborate courtship displays, and in doing so we hope to spur further research that investigates this fascinating and important area of organismal biology.

## The midbrain PAG influences motor output throughout the entire body plan

If the PAG orchestrates complex courtship displays then one critical requirement is that this brain region can activate premotor networks beyond those involved in vocal-respiratory control. This is because elaborate courtship is often multimodal, consisting of movements that utilize limb and postural musculature in a highly coordinated fashion. In support of this possibility, PAG is known to innervate multiple downstream premotor regions, a pattern of projections that could affect courtship displays (Holstege and Subramanian, 2016; Tovote et al., 2016). Furthermore, the existence of a highly conserved PAG-hindbrain circuit for the production of acoustic courtship signaling in such a wide range of taxa suggests the intriguing possibility that this circuit, while appearing to be dedicated exclusively for the production of vocalizations, might also engage musculature required for the production of non-vocal courtship signals. A combination of careful anatomical and electrophysiological studies in rodents and monkeys has indeed shown that RAm—a major premotor target of the PAG shown to be necessary for vocal production—also projects to motoneuron pools in the lumbar and sacral regions of the spinal cord that are necessary for mating behaviors (Vanderhorst et al., 2000). In females, RAm in fact projects to an entire group of motoneurons that control the lumbosacral musculature underlying female lordosis, a highly coordinated behavior that includes arching of the back to facilitate copulation (Vanderhorst and Holstege, 1995). Interestingly, variations in the projection pattern can account for species-specific variation in mating display. One example of this is the projection in hamsters to the cutaneous trunci muscles, which are necessary for tail elevation in this species’ mating display (Gerrits et al., 2000).

These mammalian studies show how the PAG-RAm circuit could coordinate a large suite of muscles in the pelvic floor and axial regions during mating behavior, and highlights the potential of the PAG to orchestrate complex muscle synergies throughout the entire body plan. Strikingly, this neurobiological theme is not limited to mammals and appears to extend throughout the vertebrate tree of life. Anatomical studies in birds have shown that RAm has similarly extensive projection patterns throughout the lower spinal cord. These projections range from the expiratory-related motoneurons in the thoraco-lumbar region (Wild, 1993; Wild and Balthazart, 2013) to motoneurons in the sacral cord that innervate the cloaca (Wild and Botelho, 2015), a sphincter muscle necessary for copulation, which also shows rhythmic contractions during mating displays and therefore may also serve as a sexual signal.

While these findings suggest that the PAG-RAm axis might constitute a fundamental brainstem module for sexual signaling — conserved throughout the course of vertebrate evolution — RAm is not the only premotor network that receives input from PAG. Other outputs can also be engaged during PAG-mediated behaviors, most clearly illustrated by the various medullary motor centers involved in locomotion and defensive responses (Tovote et al., 2016). This idea extends even to the production of courtship USVs, where tagged neurons in the PAG were shown to also project to metencephalic motor areas, such as the pons (Michael et al., 2020; Tschida et al., 2019). However, these non-RAm projections were in the minority, and it is still unclear whether PAG targets would be more diverse in more complex displays, such as those that incorporate non-vocal aspects such as limb movements and head posture.

## PAG integrates higher order contextual and motivational information

Many variables influence the initiation and continuation of display behavior. These include the social and reproductive status of a potential evaluator (receiver), the behavioral response of an evaluator to the signal, and the evaluation of potential predatory threats. It would therefore be expected that the PAG—in addition to receiving sensory signals in the visual, olfactory, tactile and acoustic modalities—might also serve as a hub for integrating information regarding the animal’s arousal state, threat risk, and perhaps even the nature of the social context in which it finds itself (e.g. rank of a mating rival, reproductive state of a potential mating partner). If it is indeed such a control center, the PAG might integrate a wide range of information to eventually commit to initiate the appropriate courtship display for the context the organism finds itself in. Consistent with this idea, anatomical studies across a wide range of vertebrates have shown that the PAG receives a confluence of inputs from higher order brain areas (O’Connell and Hofmann, 2012; Silva and McNaughton, 2019). The ventromedial nucleus of the hypothalamus (VMH) most clearly demonstrates this principle: not only is this input to the PAG itself interconnected with over 30 brain areas, but this interconnectivity appears to be conserved across all major vertebrate groups (O’Connell and Hofmann, 2011; O’Connell and Hofmann, 2012). In addition, the majority of these regions are involved in processing sociosexual information, which has led to the proposal of a Social Behavior Network (SBN) model to explain the behavioral function of this broad interconnectivity of brain regions (O’Connell and Hofmann, 2011; O’Connell and Hofmann, 2012).

Over the past few years, information has been gathered regarding how the PAG processes its inputs at a mechanistic level. Many of these studies, using targeted optogenetic approaches, have focused on inputs to PAG regions involved in the production of survival behaviors that include attack (Falkner et al., 2016; Falkner et al., 2020), escape (Tovote et al., 2016), parental behavior (Kohl et al., 2018), and mating (Inoue et al., 2019). Several recent studies have also used these approaches to investigate functional connectivity to PAG during the production of vocal courtship signals in the mouse (Chen et al., 2021; Michael et al., 2020). A common feature of the PAG to come out of this work is that it is typically kept inhibited by a dense network of intrinsic inhibitory interneurons. As such, behavioral activation can be achieved by either direct inhibition of interneurons to release PAG projection neurons from inhibition (Michael et al., 2020; Morgan and Clayton, 2005; Tovote et al., 2016) or by bypassing the inhibitory network and providing activation of projection neurons (Falkner et al., 2020). Conversely, behavioral inhibition can be achieved by direct activation of the inhibitory network as observed during the suppression of parental behavior under anxiogenic conditions (Zhang et al., 2021). In the context of USV production, activation of vocal behavior is achieved by POA neuron-mediated disinhibition of PAG projection neurons (Figure 3; Chen et al., 2021; Michael et al., 2020). Interestingly, layered on top of behavioral activation by disinhibition, PAG-projection neurons also receive direct GABAergic inputs from the central and medial amygdala. This direct input from the amygdala bypasses POA-mediated activation to directly inhibit PAG output during a threatening situation (Michael et al., 2020).

While the general architecture of PAG inputs suggests that behaviors are either activated or suppressed, inputs to the PAG might also play a role in modulating the strength of a behavioral response and perhaps even determining the type of survival behavior that is produced. Moreover, it is becoming clear that many survival behaviors are more graded than previously thought (Wei et al., 2021) and that behavioral intensity can be modulated by context (Evans et al., 2018). Based on a recent study of USV production in mice by optogenetic stimulation of PAG-projecting neurons in the POA, it appears that USV display behavior (intensity and duration), can also be regulated in a graded fashion (Chen et al., 2021). The need to regulate the intensity of display behavior as a function of context is likely to be a required feature of these control circuits. Manakins, for example, speed up their elaborate dance routines if a female is watching, compared to when there is no female around (Barske et al., 2015).

## PAG forms part of a circuit module that is hormonally regulated and well-suited to control behaviors in a reproductive context

The costs associated with courtship behavior are numerous, not only in terms of energetics but also because of predation risk inherent with the conspicuousness of the display itself. As a result, it is critical for animals to engage in these behaviors only when appropriate. The neural circuits that orchestrate and ultimately initiate courtship displays should therefore integrate information regarding breeding conditions and be regulated by circulating sex steroids. Indeed, this seems to be the case with the PAG, since it and several of its inputs (e.g. POA, VMH, amygdala) can be modulated by these hormones. Such regulation would ensure that the PAG can still provide context-specific survival behaviors such as escape and attack, while engaging in courtship displays only during the appropriate times.

Evidence that the PAG is sensitive to steroid hormone action is somewhat limited, with most support for this idea coming from work that shows that this brain area can express receptors that detect progestins (progestin receptor, or PR), estrogens (estrogen receptor, or ER), and androgens (androgen receptor, or AR). Some of the work in this regard comes from comparative studies that suggest that PAG expression of PR, ER, and AR is likely a conserved trait among mammals, birds, reptiles, amphibians, and teleost fish (O’Connell and Hofmann, 2011). More detailed studies explore the expression of these receptors in different subregions of the PAG. AR and ER, for example, can be found in the dorsal and lateral columns of PAG, as well as the ventral portions of the region (Loyd and Murphy, 2008; Murphy et al., 1999; Zheng et al., 2021). Some of this work even pinpoints likely AR and ER expression to PAG neurons that project to medullary nuclei, which in turn innervate spinal motoneurons (Loyd and Murphy, 2008). Thus, assuming expression of steroid hormone receptors does in fact confer the PAG with sensitivity to these hormones, it would seem that this brain area is susceptible to modulation by sex hormone action. Experimental support for this idea is more limited, particularly in the context of display behavior. There is work, however, that suggests in ovariectomized female doves showing that infusion of estradiol into the PAG can promotes reproductive cooing behavior (Cohen and Cheng, 1981). Other studies in midshipman fish demonstrate that the PAG contains a network of glial cells that produce aromatase, a key enzyme in the production of estrogen (Forlano et al., 2001). In this same species, both estrogens and androgens appear to act on the PAG to regulate vocal patterning (Remage-Healey and Bass, 2004). These effects occur rapidly, which means that non-genomic mechanisms of steroid action are likely at play.

Steroid hormones might also regulate inputs to the PAG as a means to influence how this latter region guides behaviors like courtship display. This idea is based on a wealth of information showing that many of these inputs are highly sensitive to sex steroid action, again expressing high levels of PR, ER, and AR (Ball and Balthazart, 2020; Feng and Neural, 2017; O’Connell and Hofmann, 2011). Specific hypothalamic inputs, for example, might carry the sensory signals from a prospective mate, and this might drive the initiation of a display (Wild, 2017). With this in mind, high levels of sex hormone receptors in these input structures might act as a gate for incoming sensory signals into the PAG, and thus regulate how these sensory signals drive courtship display. These same ideas also apply to inputs to the PAG from areas that regulate sexual motivation and arousal, such as the POA. Recent work in rodents, for example, suggests that the lateral POA contains a set of ER expressing GABAergic neurons (LPOA^ESR1^) that project directly to the PAG (Chen et al., 2021). Activation of this neuronal subset causes activation of the PAG (by disinhibiting USV-gating neurons within the PAG) and the production of USVs. Interestingly, the strength at which LPOA^ESR1^ neurons are stimulated scales with call power and duration, with low-frequency stimulation causing relatively short and lower amplitude calls while higher stimulation frequency causing calls to be louder and longer. Because stimulation within the PAG causes relatively stereotyped calls (always loud and always long) these finding suggest that LPOA can flexibly tune USV amplitude and bout length. These results parallel findings in songbirds where sex steroid activation of the POA enhances motivational components of singing that include increases in song rate, time spent singing, and song duration (Alward et al., 2013) without influencing spectro-temporal aspects of song that are more firmly rooted in motor control (Alward et al., 2018). These findings raise intriguing questions regarding the timescale over which hormonal effects can occur. Indeed, most vertebrates experience a general surge in circulating sex steroids during the breeding seasons (Goymann et al., 2007; Goymann et al., 2019), but they also often experience steroid hormone pulses on top of constitutive change (Gleason et al., 2009; Goymann et al., 2007). Both influence social decision-making processes and the production of display behavior (Fuxjager and Schlinger, 2015b; Goymann et al., 2019; Remage-Healey and Bass, 2005; Fuxjager et al., 2015c).

## PAG as a neural locus that underlies display diversification

If the PAG acts as a major coordinator of the motor programs involved in courtship display, we might also expect that this brain area plays a central role in the evolution of such behavior. Little is actually known about this topic, given that the brain’s role in generating behavioral diversity is poorly understood (Hoke et al., 2019; Jourjine and Hoekstra, 2021). This gap is even greater with respect to highly complex behavioral traits, such as display, which as we have described can evolve in a nearly endless manner. Nonetheless, identifying the PAG as a core conserved regulatory center of display output provides an important first step in identifying the neurobiological principles that might shape how display evolution unfolds. We therefore discuss this topic in the remaining sections of our review by merging properties of PAG function with tenets of evolutionary biology. Perhaps the first question to ask is the following: when might PAG evolution be associated with display design, and when might it not? We suspect that answers to this question are linked to the specific elements of the display behavior that are changing in response to selection. PAG is a likely target of selection when we see evolution in aspects of motor coordination and/or performance of intrinsic display elements. By contrast, when selection alters the motivational underpinnings of display performance, we then might suspect that upstream hypothalamic centers evolve to modify the contextual bearings of display production. Likewise, sensory processing and integration centers might also be targets of selection in cases when environmental cues and stimuli are used as triggers for display production.

There are, however, a few important points to make about when the PAG is a target of selection. First, we must recognize that PAG evolution may occur independently to promote display diversity, but it also may not. In other words, display evolution may occur through concomitant changes to all these different broad functional neural categories described above. Moreover, behavioral change may be the result of synergistic effects through these concomitant modifications, making PAG but one of many facets of display innovation. As such, PAG evolution might only support behavioral variation when it occurs alongside additional alterations to motivation, sensory processing, and performance systems. In many ways, this latter scenario makes the most sense if we consider the diversification of display behavior among species. The PAG is connected to several hypothalamic nuclei related to social behavior, and thus its capacity to coordinate movement is closely tied to these processes (O’Connell and Hofmann, 2012).

Second, we must recognize that the PAG itself is not the only site in which the evolution of motor coordination can occur. There are several other premotor nuclei in the brain and other substrates that alter basic motor functioning (Grillner and El Manira, 2020). All these loci can provide selection with access to the motor program, and thus evolve to accommodate changes to behavioral adaptation and diversification. The spinal cord is a prime example; it contains numerous microcircuits that govern suites of behavior, and temporal variation in the activation of these microcircuits confers different forms of movement (d’Avella and Bizzi, 2005). In theory, selection can alter the configuration of these microcircuits independently of the brain areas that project to them, which in turn can give rise to different forms of motor control associated with differences in display behavior (Bizzi and Cheung, 2013; Giszter and Hart, 2013). Dissecting the effects of selection directly to the PAG from those effects targeting downstream loci represents an intriguing and important challenge going forward. This issue leaves us with major questions about the nature of motor control evolution as it relates to behavior, and how selection can construct adaptive behavioral routines.

Behavioral ecologists who study display evolution have begun to tap into the importance of motor control evolution, even if they do not always approach the topic from a neurobiological viewpoint. For example, many consider display vigor as a reflection of how individuals produce a given display routine, whereas they consider display skill as a reflection of how well individuals perform this routine in natural conditions (Briffa and Lane, 2017; Byers et al., 2010; Lane and Briffa, 2022). Skill, as described here, is a rather nebulous topic, with several definitions that center around the notion of performing a ‘challenging’ display with exacting precision and/or accuracy. In many ways, it is easier to conceptualize skill from the example we introduced earlier about Olympic gymnasts. These athletes can produce a floor routine by stringing together a series of complex body movements (flips, twists, summersaults, handsprings, etc.) that few can otherwise produce because these maneuvers are so incredibly difficult for the nervous systems to generate (even with extensive practice and training) (McNitt-Gray et al., 2001). Skill is therefore the ability to perform such behavior, and it likely applies to non-human animals and the displays they use to court mates. Indeed, studies show that even simple locomotory behaviors (running, jumping) quickly become challenging to perform if they are generated more quickly (Amir Abdul Nasir et al., 2017; Wheatley et al., 2018). We speculate that the PAG could act as a direct locus for the evolution of display skill, where selection on motor skill may unfold by driving how this nucleus instantiates or possibly even coordinates this behavior.

## PAG evolution and its role in facilitating behavioral variation

Given the considerations outlined above, one might ask—how exactly could the PAG evolve to help facilitate behavioral variation among species? There are several ways for this to occur, one of which is through changes to various neuromodulator systems. There is, for example, much literature that highlights the importance of neuromodulators in remodeling brain circuits, which ultimately affects behavioral output (Nusbaum and Beenhakker, 2002). The nature of the neuromodulators that produce these effects can include hormones, including sex steroids, neuropeptides, and other more classically defined neurotransmitters. These agents often act on specific neurons to alter their intrinsic ionic properties within the circuit to impact its pattern and output (Marder, 2011). Studies that explore swimming behavior in sea slugs clearly illustrate this point, with differences in serotonergic modulation of a homologous locomotory circuit being able to drive divergent swimming patterns (Katz, 2016). Similar neuromodulators could impact how the PAG orchestrates behavioral outflow with these actions potentially altering the motor pattern to produce subtle behavioral variants, particularly since the PAG is innervated by a number of neuromodulatory areas (e.g. Raphe, LC, VTA) (Silva and McNaughton, 2019). This idea is especially attractive because even small changes to behavioral production can translate to major differences in a given behavioral trait. Indeed, many species’ display behaviors differ purely through the speed at which individuals perform singular, repetitive motor tasks (e.g. toe tapping, wing snapping, etc.) (Anderson et al., 2021a; Miles et al., 2018c; Miles et al., 2020; Ord et al., 2001; Randall, 1997).

In addition to the more classically defined neuromodulators, we also expect sex steroids (androgens and estrogens) to potentially play a role in the evolution of this region’s functionality both at the level of the PAG and its direct inputs (e.g. POA and amygdala). This idea is predicated on the point made above that describes how these regions (likely including the PAG) are known to express different levels of PR, ER, and AR (O’Connell and Hofmann, 2012), which again suggests that these brain areas are sensitive to sex steroid action. These hormones work by binding to the aforementioned intracellular receptors to induce a direct genomic effect that alters gene expression patterns, which in turn impact an organism’s behavior. Sex steroids, however, can also have non-genomic effects by acting as second messenger systems, thereby initiating functional effects within seconds or minutes of binding to a membrane-bound receptor. Numerous studies have shown that both mechanisms are at work in the brain and can influence neural functioning in a wide range of ways (Brenowitz and Lent, 2002; Remage-Healey et al., 2010; Thompson et al., 2007). From an evolutionary perspective, there is a rich literature that proposes that selection can drive the evolution of display behaviors—particularly those that are physical in nature—by adjusting the cellular mechanisms of sex steroid action in specific target tissues (Tobiansky and Fuxjager, 2020). Perhaps, the most straightforward way this phenomenon could occur in the PAG or its inputs is through the reconfiguration of ER or AR expression levels and patterns. Similarly, the levels of enzymes that produce these bioactive steroids may change in the PAG (Anderson et al., 2021b; Canoine et al., 2007; Fuxjager et al., 2015a; Johnson et al., 2018; Rosvall et al., 2012; Schuppe and Fuxjager, 2019). Our knowledge of how this might occur in the PAG, however, remains largely a mystery.

In addition to alterations to circuit properties by neuromodulators, behavioral diversity may also arise through changes to the intrinsic properties of select PAG neurons. Such changes would presumably modify the functional output of given pattern-generating circuits. Recent work in fruit flies points to this possibility, as most species within the *Drosophila* genus (which contains over a thousand different species) produce distinct courtship songs by vibrating their wings to generate acoustic display (Ding et al., 2016). These different songs are also associated with variations in wing vibration, and sometimes body display. For example, *D. simulans* and *D. mauritania*, two species which diverged approximately 240,000 years ago, show that the differences in song characteristics are largely explained by a mutation in the *slowpoke* (*slo*) locus, which codes for a calcium-activated potassium channel (Ding et al., 2016). Equally important, these studies also elucidated that natural variation of the *slo* locus affects only very specific parts of the courtship song and does not alter other key aspects of the sexual behavior and/or locomotion. Thus, sequence polymorphism of genes underlying intrinsic neuronal properties could confer highly refined and targeted changes to PAG function.

PAG evolution might also occur by rewiring events involving its circuitry. Such modifications of the neural substrate could occur locally within the intrinsic circuitry of the PAG itself, or may also occur via changes in the pattern of PAG projections to premotor targets. In some cases, changes in target connectivity can underlie surprisingly major shifts in behavioral phenotype. The nematodes provide a clear example of this process. The neuromuscular systems that govern the respective mouthparts of *C. elegans* and *P. pacificus* are comprised of the same twenty homologous neurons; however, these neurons have evolved divergent connectivity. In fact, the topology by which these neurons are connected to each other and to the oral muscular apparatus (which is also homologous between species) differs tremendously. As a result, there has been a dramatic divergence in feeding mechanics, with *C. elegans* using a pharyngeal grinder tactic and *P. pacificus* using a piercing tooth tactic (Bumbarger et al., 2013; Chiang et al., 2006; Katz, 2016). Equally intriguing work on mouse locomotion illustrates how a single mutation to the ephrin A4 (EphA4) receptor endows mice with a novel hopping gait (Kiehn, 2006; Kullander et al., 2003). Changing EphA4 function allows excitatory axons to cross the midline during development and thereby establish new connectivity with the centers that control the extensor and flexor muscles of the limbs. Flexor muscles can consequently become more synchronized, and a novel gait can emerge. Of course, the developmental processes that underlie these differences remain unclear, and they raise the intriguing question of what other components of the phenotype might change, given the pleiotropic effects of genes like those in the ephrin family (Davy and Soriano, 2005).

**Figure 4.**
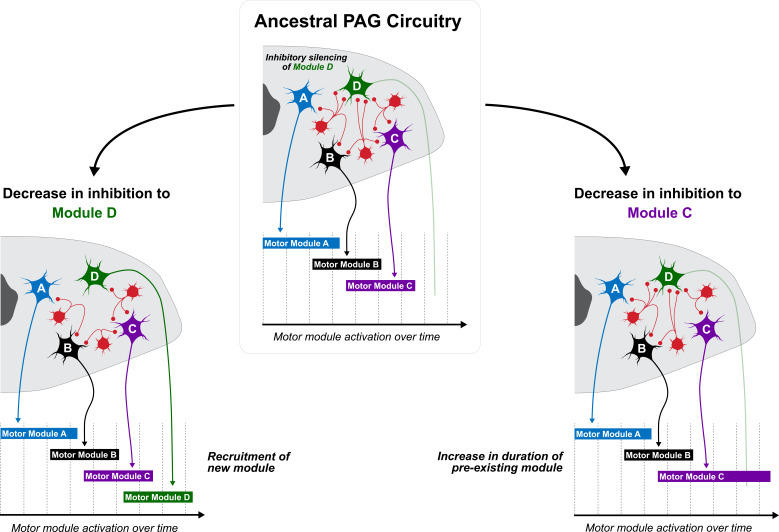
Working model for how PAG evolution might underlie display variation across species. PAG organization includes excitatory outputs (cells A-D) that are under tonic inhibition by a collection of interconnecting interneurons (red). Each excitatory cell activates a motor module, with the duration of activation corresponding to the level of inhibition that the cell experiences. In the ancestral PAG (top), cells A, B, and C receive a set level of inhibition from interneurons, which when released, activates the expression of motor modules A, B, and C, respectively, which underlie display performance. In this ancestral PAG, cell D is under high levels of tonic inhibition, and thus does not contribute to the display. However, if evolutionary pressures decrease inhibition to cell D (left), then a new module can be added to the display, thereby increasing its complexity. Likewise, if levels of inhibition on cell D remain the same but are decreased on cell C (right), then motor module D is not added to the display. Rather, motor module C is extended, and thus exaggerated within the context of display behavior.

How might the mechanisms of neural system evolution described above apply to the diversification of PAG function? We suspect that interneurons within the PAG are critical to this process. These cells tonically inhibit the PAG’s excitatory projection neurons and consequently play a major role in modifying its output. In theory, by altering the pattern by which these excitatory streams are released from inhibition, selection might influence the manner that the PAG coordinates the activation of the various motor modules underlying display (Figure 4). One way we might envision this occurring is by changing the excitable properties of these interneurons, either through modulation or differential expression of ion channels or by reconfiguring the pattern of interneuron projections within the PAG itself. It should be noted that these mechanistic possibilities would not be mutually exclusive and, in many instances, would be expected to occur simultaneously. As shown in Figure 4, one consequence of such changes could be the recruitment of additional motor modules—which are normally active in a non-courtship context—into the display repertoire. Furthermore, the activation pattern of motor modules already incorporated into the display may change. For example, decreasing the inhibition of the motor module which controls wing flapping during display might increase the duration that this module is activated, consequently increasing the time that the bird flaps during courtship. An important takeaway from this hypothetical model is that even slight changes to inhibitory properties may confer major shifts in PAG circuit output, especially given the generally sparse level of connectivity between excitatory projection neurons in the PAG. Furthermore, alterations to motor modules are not independent, in the sense that modifying one movement may lead to changes in the patterning of muscle synergies which constitute a display sequence. This point is nicely illustrated by work in frogs, which shows that walking, jumping, and swimming are largely controlled by the same suite of muscle synergies (i.e. coherent activational patterns of discrete groups of muscles through space and time), despite being markedly different forms of locomotion. Indeed, differences in these movements are attributed to one or two behavior-specific muscle synergies (d’Avella and Bizzi, 2005). This work is intriguing in the context of motor control for display, because it suggests that one straightforward change to the activational program of muscle suites associated with a given movement can ripple out and affect other elements of a display routine, which in turn could alter the entire courtship performance and aesthetic. In short, we speculate that altering the inhibitory dynamics of PAG interneurons may provide ample raw material for generating display diversity. As a result, these mechanisms might approximate how sexual selection has acted upon the intrinsic circuitry of the PAG to drive the evolution of courtship display in vertebrates.

## Evolution of behavioral complexity by modification of “higher-order” inputs to the PAG

Interplay between the PAG and its upstream inputs may provide a critical route by which display routines evolve, especially for the more complex displays that demand higher-order coordination or processing (e.g., learning). As described above, a defining feature of the PAG is that it acts as an information integrator receiving inputs from a large number of higher-order brain areas including from dedicated neural circuits within the telencephalon (see Silva and McNaughton, 2019 for a comprehensive review of PAG connectivity). It is relatively easy to imagine, therefore, how such inputs might interact with PAG to ultimately drive or shape the elaborate motor programs of courtship display. Although the vast majority of species studied do not necessarily have an intricate ‘display control system’, this idea can be illustrated by thinking about how the complex acoustic patterns in birdsong are shaped by the ‘song system’, a specialized forebrain neural circuit critical for the production of learned vocalizations. While this illustration focuses primarily on a bottom-down scheme – the PAG contains very few efferent projections to forebrain (with the exception of hypothalamus) – given the recurrent nature of many circuits (Thompson and Swanson, 2010), we recognize that the PAG could also play an indirect role in shaping higher order circuits (Ashmore et al., 2008; Hamaguchi et al., 2016).

In songbirds, as in many other species (Chen et al., 2021; Holstege and Subramanian, 2016; Jürgens, 1994; Seller, 1981; Tschida et al., 2019), stimulation of the PAG has been long known to evoke call vocalizations, while lesions can completely eliminate this behavior. In zebra finches, females produce acoustically simple ‘harmonic stack’ contact calls that show significant variability in duration. By contrast, males produce contact calls that are acoustically more complex and highly stereotyped in their spectro-temporal properties (Simpson and Vicario, 1990; Williams, 2008). Neural recordings from the ‘song system’, including this system’s output structure, known as nucleus RA, reveal that neurons are active during the production of male calls in finches (Ma et al., 2020; Ter Maat et al., 2014) and that targeted lesions to these areas completely eliminate the fine acoustic features observed in male calls essentially transforming them into female-like calls (Fukushima and Aoki, 2000; Simpson and Vicario, 1990).

Detailed neurophysiological recordings from RA, which shares many functional properties with motor cortex (Colquitt et al., 2021), in male songbirds during singing show that its neurons produce a highly precise sparse neural code (Chi and Margoliash, 2001; Leonardo and Fee, 2005) that surprisingly cannot be linked directly to the observed acoustic features of individual song syllables (Sober et al., 2008). This highly abstract code therefore likely needs to be transformed into the specific motor commands that can be read by motoneurons. We speculate that the PAG, a portion of which receives a major projection from RA, might be a critical node in this transformation. Going back to the male call example, these findings are consistent with the idea that PAG implements a basic ‘call plan’ and that this plan is sculpted by descending inputs from the ‘song system’ acting to constrain call duration and adding acoustic feature complexity. We might suspect that the PAG plays a similar role in the context of singing. This idea is speculative, but it provides an intriguing example of how higher order inputs might be able to shape the features produced by PAG circuits. Surprisingly, the exact role that the RA-recipient PAG module, known as DM (Kingsbury et al., 2011), plays in song production has never been directly tested (Haakenson et al., 2020).

While the above scenario might suggest that these higher order inputs to the PAG could possibly act as targets of selection for song evolution, such arguments might generally be dismissed because (i) song is learned and (ii) the ‘song system’ neural code acquires its specific features during song ontogeny, which are shaped by both social and environmental experience. Interestingly, recent work suggests that certain temporal features of song might in fact be encoded in distinct nuclei within the ‘song system’ and be under hereditary control (Mets et al., 2021). The degree to which genetic predisposition and culture might interact to affect song control functionality—and how they impact regions like the PAG—is unclear, but remains an exciting line of future research (Fehér et al., 2009).

## PAG as a source of evolutionary constraint or potentiation

Because the PAG is likely involved in the evolution of display behavior, it also likely has the capacity to constrain and/or promote the diversification of different courtship display routines. The role of the nervous system in this facet of behavioral evolution (let alone display evolution) is remarkably poorly understood, yet several studies do suggest that motor control of behavior provides a major hurdle to the fundamental properties of its design (Katz, 2011; Katz and Hale, 2017). Thus, we must recognize that certain PAG phenotypes might limit how behavioral novelty can (or cannot) arise, or in some cases these phenotypes may promote display innovation. Take the mouse locomotion example described above as a conceptual example—when the point mutation to *EphA4* arises, a novel gait emerges in the population (Jung and Dasen, 2015; Katz, 2016). With this novel gait, of course, comes new opportunities for selection to drive the evolution of display behavior (assuming the novel gait is not extinguished by purifying selection). We may therefore think of the ancestral locomotory phenotype, which is born through a canalized neuro-developmental process linked to the functionality of the ephrin system, as a source of constraint that limits gait development. But, by changing how the ephrin system works during development, the population breaks free of this constraint and acquires new behavioral abilities. Presumably, the endowment of these novel locomotory skills incur their own set of constraints, even if they simultaneously confer new opportunities for selection (Miles et al., 2020).

**Figure 5.**
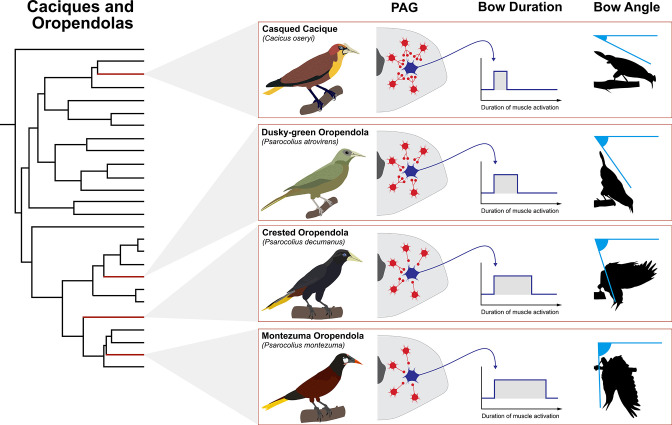
Theoretical model of how PAG evolution might shape diversification of courtship bow display in New World blackbirds (family: Icteridae). Shown is the phylogeny of the oropendolas and caciques (Powell et al., 2014) with representative species highlighted by red branches. These taxa all perform bowing displays, but the angle at which they bow differs markedly. In theory, these differences may be related to the degree of tonic inhibition of excitatory streams that flow from the PAG. Greater inhibition (e.g. casqued cacique) is denoted by more red interneurons and more synaptic terminals projecting onto the blue excitatory neuron that leaves the PAG. This results in shorter duration muscle activation and thus a lower bow angle. By contrast, less inhibition (e.g. Montezuma oropendola), denoted by fewer red interneurons and fewer synaptic terminals projecting on to the blue excitatory neuron, results in extended muscle activation and greater bow angle. Indeed, Montezuma oropendolas dangle upside-down from their display branch when they perform their bow (Miles and Fuxjager, 2018b), which is by far the most elaborate form of this behavior in the family (Miles et al., 2017). It is no surprise that this species is also under especially strong sexual selection, relative to the other taxa within this small clade. Bird illustrations by Ryan Schwark.

Studies are needed to address this possibility in the PAG. However, we suspect that signatures of PAG constraint/potentiation can be seen through the phylogenetic reconstruction of different species’ display routines. New world blackbirds (family: Icteridae) could provide an accessible example of this phenomenon. Species in this diverse family are found across most of North, Central, and South America, and they show an incredibly wide range of sociosexual displays that arise through sexual selection by either male-male competition or female choice (or both) (Jaramillo and Burke, 1999). These displays are wildly different, with some species performing highly complex flight and postural displays and other species performing simple gestures (Miles et al., 2017). Many species of New World blackbird have even dispensed with physical courtship display altogether (Price et al., 2007). Yet, one of the most remarkable features of these displays is that particular maneuvers appear to show some degree of conservation. Oropendolas and caciques, for instance, often exhibit bowing displays, in which males bob their heads downward (Miles et al., 2017; Price and Lanyon, 2002). The different taxa that make up this clade show extraordinary variation in the extent of this bow (Figure 5), with some species performing a slight bow and other species performing an extreme bow in which they dangle upside-down from a tree branch (Miles and Fuxjager, 2018b; Price et al., 2006). By contrast, grackles perform a very different display that contains basic patterns of feather-ruffing, wing-drooping, and billpointing behavior (Ficken, 1963; Post and Wiley, 1992; Wiley, 1976). Grackles also tend to vary the duration of these different display elements, with some performing much longer displays than others. Thus, we again see behavioral evidence of a conserved motor program that underlies the basic elements of the display, with variation occurring in terms of how the different display maneuvers are dispatched through time.

With respect to the PAG, we might expect that it evolves innovations in the circuitry that coordinate the motor subroutines that comprise an entire display. Figure 5 highlights how this might occur by showing different hypothetical configurations of the PAG which underlie different durations of display bowing. As an example, circuitry that confers greater inhibition of excitatory motor modules that underlie bowing may result in a briefer bow that does not extend very deep. However, less tonic inhibition of these excitatory motor modules may underlie a longer bow, which results in the bird swinging all the way upside-down. Such ideas are completely speculative at this point, but they are important because they begin to paint a picture of how brain systems might evolve to drive display diversification. To this end, we might even begin to think about using phylogenetic comparative methodology to pinpoint in a phylogeny (such as the one that shows the relationships among New World blackbirds) when display innovation likely emerged, and in doing so we can potentially highlight ancestors in which novel PAG configurations might have liberated the species from motor constraints on display design. Presumably, when such evolutionary events occur, they create new opportunities for selection to drive the evolution of courtship behavior. Thus, variation in these motor programs might subsequently emerge when selection begins to alter temporal patterns of behavioral execution, another feature that could easily be regulated through PAG function. Notably, this model is completely agnostic to how the PAG might change to facilitate the evolution of motor programming as it relates to display divergence. As we describe above, such effects can occur through discrete changes to PAG modulation and/or rewiring, as well as changes to the upstream input to the PAG (or any combination of the three).

Building on this idea, we must recognize the possibility that modification to a motor program held within the PAG can indirectly affect other neural processes associated with behavioral control, and these effects can also influence display performance. Oropendolas and caciques that engage in extreme bowing behavior, for example, may trigger other reflexive responses that are associated with a loss of balance. In doing so, the brain may incorporate the production of different corrective movements that change the gestural and/or postural composition of the routine. Such effects may alter the visual aesthetic of the display, and thus create new opportunities for selection. Importantly, this hypothetical example shows how one single change in the PAG could confer a behavioral chain reaction that fundamentally alters display performance. Thinking about behavioral elements in a courtship display as indirect byproducts that emerge through adaptive functional synergy of the nervous system is an intriguing way to imagine how display diversity emerges, but to date, has seldomly been studied.

We recognize that these ideas are largely speculative and based only on behavioral data. Large-scale comparative studies are needed to connect PAG phenotype to behavioral variation within and among species. Even straightforward neuroanatomical studies would be informative, potentially revealing clues about how PAG design might support the processes underlying display adaptation and diversification. Moreover, we are currently experiencing an intellectual invigoration with respect to phylogenetic comparative methods (Harmon, 2019; O’Meara, 2012; Pennell and Harmon, 2013), which can be combined with studies of neurobiology to trace behavioral evolution to its macro-and microevolutionary origins (Jourjine and Hoekstra, 2021).

## PAG evolution and the origination of courtship displays

If the PAG acts as a key coordinator of courtship display, then it also likely helps reveal some of the mysteries associated with the origins of these displays. As we describe above, one of the fundamental ideas about the origination of display behavior centers on the notion of ritualization (Bradbury and Vehrencamp, 2011; Lorenz, 1966; Tinbergen, 1952). In effect, ritualization is the process by which selection for display behavior proceeds through the modification of existing (ancestral) behavioral program, which otherwise has nothing to do with communication (Scott et al., 2010). From a neurobiological perspective, this means that display evolution likely occurs through modifications to neural systems that underlie these rudimentary behavioral routines. We therefore suspect that the PAG itself could be one of the primary neurobiological substrates involved in this process, an idea largely attributed to the fact that this area holds many of the motor programs for basic survival behaviors that include escape, attack, and respiratory functions (Subramanian et al., 2008). Thus, if ritualization works by amplifying and/or exaggerating specific elements of these programs, then changes to the PAG should be a parsimonious way for this to happen.

A good example can be found in the preparatory movements of birds, which are the basic postural movements made before flight. When, for instance, birds like gannets, boobies, darters, or cormorants get ready to take off, they assume a low crouch, while pointing their heads upward and getting their wings ready to beat. At this same time (lift-off), birds also finely adjust their breathing, so that it is integrated appropriately with their wing-beats during powered flight (Berger et al., 1970). It turns out that many of these preparatory movements have been ritualized into exaggerated postural displays used for courtship (Kennedy et al., 1996). Thus, the PAG likely plays a role in controlling the coordinated movements of the legs, neck, and wings to help an individual prepare to take off, while also adjusting respiration accordingly. Strong sexual selection can therefore drive the evolution of this intrinsic program to lengthen the duration of specific movements and/or amplify their magnitude. As we describe above, even seemingly small changes to the functional properties of the PAG could confer outsized changes to behavioral output, at least from our own aesthetic evaluation of the display as it is produced.

There are several other examples of ritualization in the animal kingdom, many of which can hypothetically arise by way of selection acting through the PAG to alter core movement programs that otherwise play no role in communication. Some frog species, for example, perform waving displays with their hindlimbs (called foot-flags), which are thought to have evolved as ritualized reflexive leg kicks that males originally used to fight rivals (Preininger et al., 2013). Another well-established example of ritualization occurs in ducks, where elaborate courtship postures used to advertise colorful plumage likely evolved through a ritualization of preening behavior (Lorenz, 1941). Importantly, many of the relatives of these different species perform displays that seem like they are in intermediate stages of the ritualization process, creating a potentially compelling way to look at how the PAG might be involved in the evolution of non-communicative motor patterns into communicative ones. Again, in both of these cases, the behavioral traits being ritualized are likely coordinated with respiratory programs, adding further support to the idea that the PAG is the likely substrate through which ritualization occurs.

Unfortunately, few biologists are currently exploring the neurobiology of ritualization. This dearth of information is somewhat surprising given that ritualization itself is a description of a neurobiological transition. Still, we suspect that the intrinsic structure and function of the PAG may endow evolution with the raw material to drive ritualization. This should create an urgency to uncover how the PAG functions and how its programming relates both to display routines and their supposed precursors. Such research would promise to fundamentally advance our understanding of how display evolution can and cannot occur.

## Conclusions and future directions

We propose that evolution of elaborate courtship display often occurs by way of interactions between sexual selection and the PAG. Indeed, this midbrain nucleus is known for being critical in the production of instinctive survival behaviors, and thus it is well placed to co-opt these motor patterns through the process of ritualization into the complex motor patterns that are typically observed during courtship displays. The PAG, which is highly sensitive to sex steroids, also receives a large number of inputs from hypothalamic and other areas that are themselves highly sensitive to these hormones and that are known for their role as regulators of sexual arousal and motivation. Accordingly, we suspect that the PAG helps integrate this information into the motor programs that manifest as the unusual and extraordinary display routines that courters (signalers) perform to mediate the dynamics of mate choice. We, of course, do not expect that the PAG is the only locus that evolves in a way to facilitate the modification and diversification of such behavior, and thus we have highlighted how other parts of this conserved circuit module might mediate the way display routines change over evolutionary time. Nonetheless, we believe that the PAG is a key player in this process, and it merits significant attention from researchers who are interested in discovering how neural systems evolve to accommodate behavioral innovation and expression. With these points in mind, we conclude our review by focusing on several future research directions that neurobiologists, animal behaviorists, and evolutionary biologists should consider going forward.

First, it will be important to establish the PAG’s role in a broader range of display behaviors. This should involve not only vocal displays, but also the other important aspects of body movement that are integrated into courtship routines. As we describe above, gesture, posture, and movement are fundamental to many species’ display behavior. One of the more intriguing questions is how different motor modules within the PAG might be integrated to produce complex multimodal displays. Here, we could leverage species that produce displays that incorporate different modalities, depending on the context in which they are produced. Certain species of New World blackbirds (family: Icteridae), such as the brown-headed cowbird, produce an exaggerated wing display when singing to males, but largely suppress this display when singing to females (O’Loghlen and Rothstein, 2010). Differential context-dependent expression of vocal and postural displays should allow for identification of the discrete PAG modules that activate each behavior, while opening the possibility for dissecting these circuits using targeted optogenetic tools. It should also be possible to leverage the arsenal of mouse genetics to address similar questions given that they often combine USVs with a broad suite of movement patterns (tail movements, ear wiggling, hopping, darting, etc.) (Gonzalez-Flores et al., 2017; Hull and Dominguez, 2014; Sachs and Barfield, 1976). A recent exciting study investigating the role of PAG in the stereotyped predatorial hunting sequence in mice suggests this is possible. Specifically, using a combination of electrophysiological and optogenetic approaches, Yu et al., 2021 show that distinct populations of neurons in the dorsolateral PAG are associated with each of the three actions in this behavior (chase, attack, and eating) and that these populations are sequentially activated during hunting (Yu et al., 2021). Such studies would require behavioral standardization across labs, as well as closely related species, to assess how these seemingly ‘micro behaviors’ might be sequenced to form a more complex ritualized display.

In this same vein, researchers should begin to investigate the role of the PAG in other species’ display maneuvers that rely on complex body movement. This is a challenging task, but advances in the ability to manipulate and monitor neural circuits is making it more possible to do so (Fuxjager et al., 2017; Spool et al., 2021). In the spirit of this goal, there are also several core approaches to studying the PAG and display that biologists can leverage; for example, comparisons of sex differences in PAG structure and function are possible and easy to do, especially when species show clear sex differences in courtship behavior. Similarly, seasonal changes in PAG function that might relate to seasonal differences in the ability to perform display routines are another starting point.

Second, although it may seem like nearly all displays studied at the brain-level rely in part on PAG engagement, there are some that may not. Courtship calls in *Xenopus* frogs, for example, appear to engage descending neural pathways that bypass the PAG (Barkan et al., 2018). We do not know why this is the case, but one possibility centers on the idea that *Xenopus* do not use air movements to drive vocalizations like most other taxa, including most frogs and toads (Kwong-Brown et al., 2019). Thus, integration between display behavior and respiration may be a critical prerequisite for PAG involvement in the coordination of display motor programming, which likely occurs in the vast majority of terrestrial taxa. Interestingly, afferents from the central amygdala (CeA), a structure that when stimulated causes vocalization in *Xenopus*, has dense afferent projections to the PAG (Ballagh, 2014) suggesting that the PAG might play a key, but still undetermined, role in coordinating acoustic vocalizations in this species. Few experiments have rigorously attempted to address this possibility. Regardless, studies that explore taxonomic anomalies with respect to PAG and display behavior may shed light on the principles that describe how motor control systems relate to behavioral evolution.

Third, the role of the brain—specifically the PAG—in the origination of display behavior must be investigated. How do new display routines arise, and how are existing routines diversified at the level of the nervous system? As we highlight in our review, answers to these questions evoke the concept of ritualization to describe the process by which existing behaviors are transformed into exaggerated displays that have a clear communicative function. Yet, ritualization is seldom, if ever, studied in the context of neurobiology, and this gap has created an enormous blind spot in our understanding of signal design. If research can identify the PAG as the neurobiological origin of display behavior, then a host of other questions regarding mechanisms of display diversification and elaboration can be subsequently asked. Likewise, such work would open the door to research that explores the neural basis of evolutionary constraint on signal design.

In sum, our knowledge of elaborate animal displays has grown tremendously in recent decades through a large number of pioneering behavioral studies that are unafraid to investigate such behavior in a wide range of taxa, including several exotic species. The spectacular diversity of these displays has come fully into view, and it is now time to bridge the gap between the tremendous behavioral and evolutionary work in this area of organismal biology with the field of modern neurobiology. Such integration promises to uncover important key mysteries about the brain’s role in behavioral evolution and diversification that many have long sought to understand.
